# ABC transporters in fish species: a review

**DOI:** 10.3389/fphys.2014.00266

**Published:** 2014-07-22

**Authors:** Marta Ferreira, Joana Costa, Maria A. Reis-Henriques

**Affiliations:** CIIMAR/CIMAR - Interdisciplinary Centre of Marine and Environmental Research, Laboratory of Environmental Toxicology, University of PortoPorto, Portugal

**Keywords:** multixenobiotic resistance, efflux proteins, fish, detoxification, regulation, biotransformation

## Abstract

ATP-binding cassette (ABC) proteins were first recognized for their role in multidrug resistance (MDR) in chemotherapeutic treatments, which is a major impediment for the successful treatment of many forms of malignant tumors in humans. These proteins, highly conserved throughout vertebrate species, were later related to cellular detoxification and accounted as responsible for protecting aquatic organisms from xenobiotic insults in the so-called multixenobiotic resistance mechanism (MXR). In recent years, research on these proteins in aquatic species has highlighted their importance in the detoxification mechanisms in fish thus it is necessary to continue these studies. Several transporters have been pointed out as relevant in the ecotoxicological context associated to the transport of xenobiotics, such as P-glycoproteins (Pgps), multidrug-resistance-associated proteins (MRPs 1-5) and breast cancer resistance associated protein (BCRP). In mammals, several nuclear receptors have been identified as mediators of phase I and II metabolizing enzymes and ABC transporters. In aquatic species, knowledge on co-regulation of the detoxification mechanism is scarce and needs to be addressed. The interaction of emergent contaminants that can act as chemosensitizers, with ABC transporters in aquatic organisms can compromise detoxification processes and have population effects and should be studied in more detail. This review intends to summarize the recent advances in research on MXR mechanisms in fish species, focusing in (1) regulation and functioning of ABC proteins; (2) cooperation with phase I and II biotransformation enzymes; and (3) ecotoxicological relevance and information on emergent pollutants with ability to modulate ABC transporters expression and activity. Several lines of evidence are clearly suggesting the important role of these transporters in detoxification mechanisms and must be further investigated in fish to underlay the mechanism to consider their use as biomarkers in environmental monitoring.

## Introduction

The maintenance of the overall environmental health is quite dependent on aquatic ecosystems. Species overexploitation, pollution from urban, industrial and agricultural areas, as well as habitat loss and alteration have been contributing to the decline of aquatic environmental health. Toxic heavy metals resultant from industrial processes, persistent organic pollutants (POPs) from agricultural and industrial sources and polycyclic aromatic hydrocarbons (PAHs) resultant from oil spills are among the sources of major concern regarding water pollution, due to their known toxic effects on the aquatic biota (Van Der Oost et al., 2003; Weber et al., 2008). More recently, a highly diverse group of contaminants, denominated emergent contaminants (EC), substances that are detected in the environment but are not included in regulatory monitoring programs are added as potential threats to ecosystems and human health and safety (Santos et al., 2010; Celander, 2011). They encompass a large number of compounds that include pharmaceuticals, hazardous and noxious substances (HNS), personal care products (PPCPs), nanomaterials, among others (Farré et al., 2008). Some of these EC have been receiving attention, since these compounds are continually introduced into the aquatic environment as complex mixtures via a number of routes, but primarily by both untreated and treated sewage (Daughton and Ternes, 1999). In order to survive, living organisms have developed strategies of protection to the adverse effects of pollutants. Chronically exposed organisms use a well-developed detoxification mechanism in order to reduce the toxic effects of pollutants (Van Der Oost et al., 2003). These mechanisms include the activity of specific proteins to preclude the permanence of toxic compounds or their metabolites in the cells - some members of the ATP-Binding Cassette (ABC) superfamily—and enzymatic systems to transform the chemicals into a more easily excreted form—phase I and phase II biotransformation enzymes. Thus, the knowledge of the functionality of these detoxification pathways is of critical importance, to support the availability of the aquatic resources for future generations.

The main points of this review will be the detoxification mechanisms adopted by fish species, focusing in (1) the regulation and functioning of ABC transporters; (2) the cooperation with phase I and II biotransformation enzymes; and (3) the ecotoxicological relevance of emergent pollutants with ability to modulate ABC transporters expression and activity. Recent studies have raised the possibility of a coordinated action between these groups of proteins, resulting in a powerful and effective mechanism of cellular detoxification.

## The superfamily of ABC efflux transporters

The ABC genes represent one of the largest family of transmembrane transporter proteins encoded in the human genome (Dean et al., 2001). These proteins bind to ATP and use that energy to drive the transport of a wide variety of molecules across cellular membranes (Table 1) (Dean and Annilo, 2005). So far, 58 members of the ABC family have been described, including 49 human ABC genes and 9 additional genes found in other animal species. From the 58 genes, 68% are present in all vertebrate genomes, suggesting that their structure and functions have been largely conserved throughout the evolution of vertebrate species (Dean and Annilo, 2005). Based on the sequence and the organization of the ATP-binding domains, also known as nucleotide binding domains (NBDs), ABC proteins were grouped into eight subfamilies in eukaryotes (A–H), with seven of these (A–G) present in the human genome (Dean and Annilo, 2005), as shown in Table 1. The ABCH subfamily has been identified so far only in zebrafish and the function is still unknown (Popovic et al., 2010).

**Table 1:** **List of known ABC genes, functions and number of members found in human and zebrafish genomes**.

**Subfamily**	**Members**	**Functions**	**Human**	Zebrafish^*^
*ABCA*	*ABCA1 to ABCA13*	Cholesterol efflux, phosphatidil choline efflux, N-retinylidiene-PE efflux	12 members	7 members
*ABCB*	*ABCB1 to ABCB11*	Peptide transport, iron transport, Fe/S cluster transport, bile salt transport, xenobiotics transport	11 members	9 members
*ABCC*	*ABCC1 to ABCC13*	Organic anion efflux, nucleoside transport, chloride ion channel, sulfonylurea receptor, potassium channel regulation, xenobiotics transport	13 members	11 members
*ABCD*	*ABCD1 to ABCD4*	Very long chain fatty acids transport regulation	4 members	4 members
*ABCE*	*ABCE1*	Elongation factor complex	1 member	1 member
*ABCF*	*ABCF1 to ABCF3*	Unknown function	3 members	3 members
*ABCG*	*ABCG1 to ABCG5*	Cholesterol transport, sterol transport, toxin transport	5 members	5 members
*ABCH*	*ABCH1*	Unknown function	No members	1 member

* *These genomes are incompletely assembled and annotated and the gene numbers may be higher. Adapted from Dean and Annilo (2005)*.

**Figure 1:**
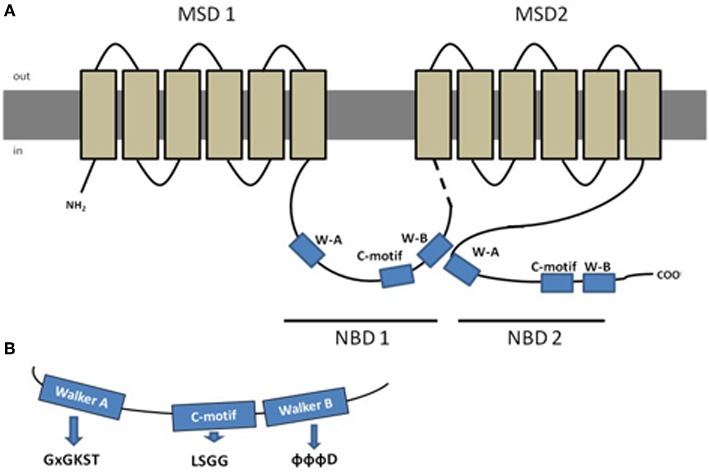
**Schematic representation of a typical ABC protein and characteristic amino acid sequences found in Nucleotide Binding Domains (NBDs). (A)** Lipidic bilayer is shown in gray, membrane spanning domains (MSDs) in light brown and nucleotide binding domains (NBDs) in blue, dashed line represents the linker region; **(B)** aminoacid sequences characteristic from the conserved domains Walker A (x represents any aa), C-motif and Walker-B (Φ represents hydrophobic residues). Adapted from Dean et al. (2001), with permission.

Typically, a functional protein contains two NBDs and two membrane spanning domains (MSDs), the latter being composed by 6–10 membrane spanning α-helices that confer the substrate specificity (Figure 1) (Szakács et al., 2008). Eukaryotic ABC proteins are organized either as full transporters (containing two NBDs and two MSDs), or as half transporters (containing one NBD and one MSD), that have to form homo- or heterodimers in order to constitute a functional protein (Dean et al., 2001). Some variation exists in protein structure in the different subfamilies, as it will be described further ahead, but a high degree of structural and sequence homology is shared among all ABC transporter proteins (Linton and Higgins, 2007).

### Multidrug resistance (MDR) associated ABC transporters

Juliano and Ling (1976) described a multidrug resistance (MDR) phenomenon, found in tumor cell lines of mammals, as the result of low intracellular accumulation of anti-cancer drugs, and related it with the overexpression of a transmembrane protein, responsible for an ATP-dependent efflux of those drugs into the extracellular medium. This protein, denominated as Permeability glycoprotein (Pgp) and encoded by the *ABCB1* gene, was the first ABC transporter to gain importance and later to be related to cellular detoxification. From this point, several other members of the ABC transporter efflux family, other than ABCB, have been identified as capable of interacting with endo- or xenobiotic compounds, including members of the ABCC and ABCG subfamilies (Cole and Deeley, 1998; Costa et al., 2012b; Fischer et al., 2013; Ferreira et al., 2014). Due to their ecotoxicological role these three subfamilies of transporters will be addressed in more detail in the following sections.

#### P-glycoprotein (Pgp)

**Figure 2:**
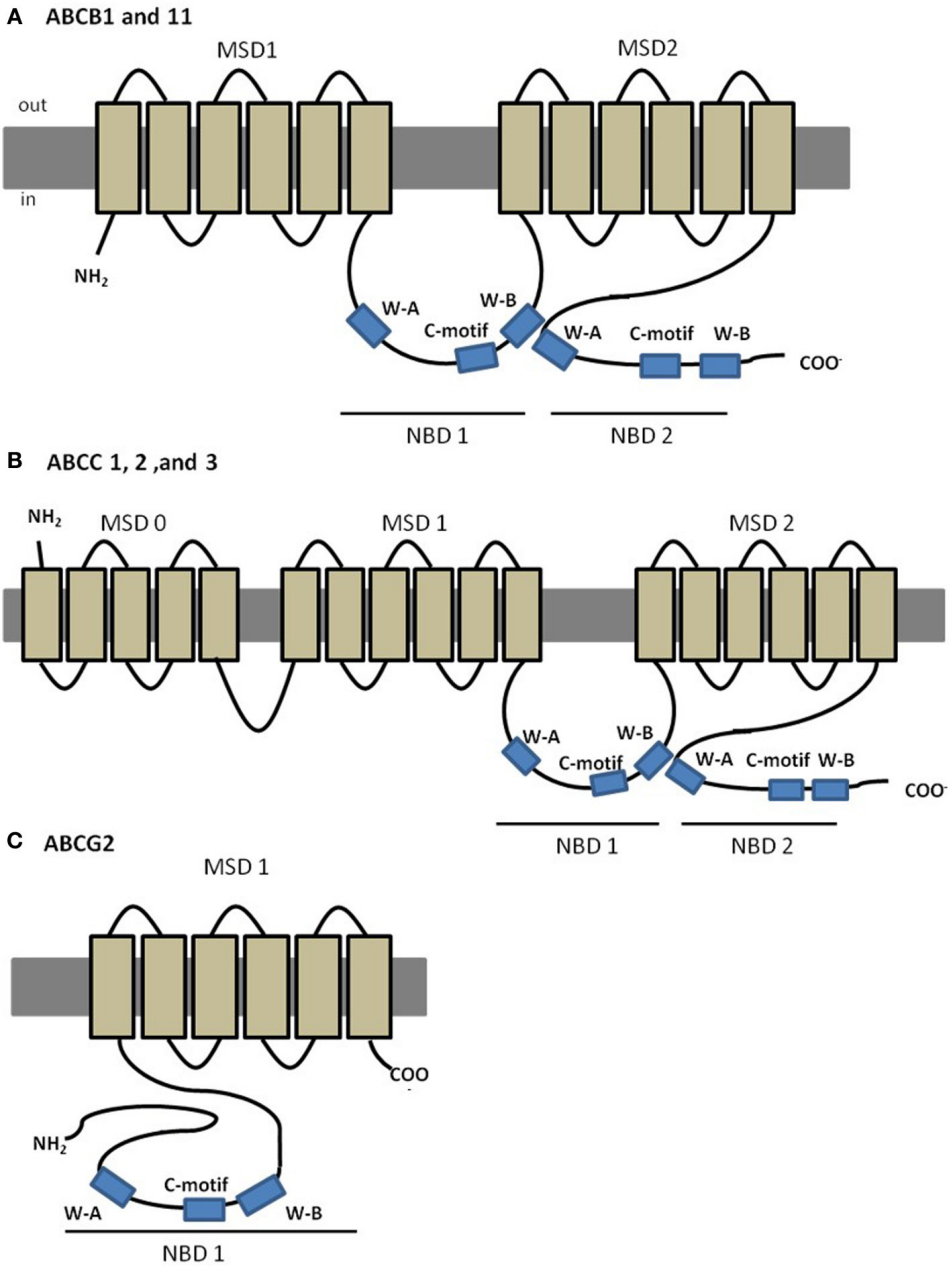
**Predicted structures of MDR associated members of ABC transporters. (A)** Predicted structure of ABCB1 and ABCB11; **(B)** predicted structure of “long-chain” ABCC proteins, ABCC1, 2 and 3; **(C)** predicted structure of ABCG2.

The best characterized ABC transporter is Pgp (subfamily B, member 1: ABCB1; MDR1). In humans, Pgp is encoded by two different isoforms of the gene (MDR1 and MDR3); the class 1 has been implicated in drug resistance whereas the function of class 3 isoform is still unknown (Georges et al., 1990). ABCB1 (MDR1) was the first eukaryotic ABC member identified as results of its implication in MDR of cancer cells to chemotherapy (Gottesman and Ling, 2006). Further evidence of its MDR abilities included decreased drug accumulation in cells transfected with *ABCB1* gene (Ueda et al., 1987), and increased drug accumulation in gene knockouts organisms, compared to the wild-type organism (Schinkel et al., 1994). In humans, ABCB1 is a 170–180 kDa protein containing ~1280 amino acids, with a predicted four-domain structure, typical of most eukaryotic ABC transporters, with two NBDs each preceded by a MSD composed of six transmembrane (TM) helices (Loo and Clarke, 1999) (Figure 2A). MDR provided by Pgp is a consequence of its remarkable non-specificity with respect to the substrates. Several researchers have focused their attention on the understanding of the promiscuity of this transporter regarding its substrates, and have indicated common characteristics among them, such as moderate hydrophobicity, small size and positively charged or neutral domains, and include natural products, chemotherapeutic drugs or steroids (Litman et al., 2001; Higgins, 2007; McDevitt and Callaghan, 2007). Pgp can also interact with modulators that are able to reverse MDR by blocking or saturating Pgp binding locations, called chemosensitizers.

The sister of P-glycoprotein (Spgp, ABCB11, BSEP), follows the same predicted structure as ABCB1 (Figure 2A), and studies in rats showed predominant expression in liver, canalicular localization, and high affinity to transport primary and secondary bile salts (Gerloff et al., 1998). The role of ABCB11 in drug disposition, if any, is considerably more limited than that of its close relative, Pgp.

#### MRPs—multiresistance associated proteins

After the discovery of ABCB1, the study on cancer cells displaying MDR phenotype not associated with ABCB1 expression, led to the discovery of ABCC1, the founding member of the ABCC subfamily (Cole et al., 1992). So far, this subfamily includes a total of 13 members, most of which are active ATP-dependent membrane transporters for organic anions of therapeutic compounds (Honorat et al., 2009). Among its members, at least five (ABCC1, ABCC2, ABCC3, ABCC4 and ABC5) are potentially involved in mediating drug resistance (Cole et al., 1992; Kool et al., 1997; Evers et al., 1998). Members of the ABCC subfamily can fall in one of two different subclasses, “short” and “long” (Kast and Gros, 1997; Bakos and Homolya, 2007; Honorat et al., 2009). The so called “long” ABCC transporters (ABCC1, 2, 3, and 6) present an additional N- terminal MSD (MSD0), of approximately 250 amino acids, a unique feature of these specific transporters (Figure 2B) in comparison to the “short” transporters. ABCCs are ~190 kDa proteins, and share 14–25% amino acid identity with ABCB proteins (Cole et al., 1992; Keppler and Konig, 1997). From the ABCC subfamily, ABCC1 and ABCC2 are the best characterized transporters with existing evidences from animal models to have a role in organ defense, while other members like ABCC3, 4, and 5, are far less studied (Mayer et al., 1995; Keppler and Konig, 2000).

#### BCRP—breast cancer resistance protein

The second member of the ABCG subfamily, ABCG2, is a ~72 kDa efflux transporter, whose overexpression in permanent cell-lines has been associated with high levels of resistance to a variety of anticancer drugs, such as mitoxantrone, doxorubicin, and daunorubicin, without evidences of expression of the well-characterized genes for ABCB1 or ABCCs therefore contributing to a MDR phenotype. This protein is also known as breast resistance associated protein (BCRP) (Doyle et al., 1998; Doyle and Ross, 2003), mitoxantrone-resistance protein (MXR) (Miyake et al., 1999) or placenta-specific ABC protein (ABCP) (Allikmets et al., 1998) since it was cloned independently by 3 different groups. Members of the ABCG subfamily have a unique domain organization, since, unlike the remaining subfamilies, these are half-transporters, composed by one single NBD followed by one MSD (Figure 2C). In addition, they also present a unique protein configuration, in which the NBD precedes the MSD, whereas ABCBs and ABCCs have an opposite domain arrangement, that is, the MSD is followed by the NBD (Figure 2C).

Functional characterization studies have demonstrated that ABCG2 can transport a wide range of substrates, from chemotherapeutic agents to organic anion conjugates (Mao, 2005). Moreover, it seems that ABCG2 has higher affinity to transport sulfated conjugates of steroids and xenobiotics over GSH and glucoronide metabolites (Chen et al., 2003).

## From MDR to MXR—ABC efflux transporters in aquatic organisms

Aquatic organisms are able to survive and thrive in heavily polluted environments, showing surprisingly low accumulation of pollutants in body organs (Kurelec and Pivčević, 1989). Kurelec and co-workers were the first to demonstrate that aquatic organisms adopt strategies of xenobiotic transport, in order to improve adaptation to pollutants in their habitats (Kurelec and Pivčević, 1989, 1991; Kurelec, 1992). To this phenomenon of resistance, Kurelec coined the term of multixenobiotic resistance (MXR) (Kurelec, 1992), and identified its biochemical basis as similar to the one adjacent to the MDR phenotype. The presence of a drug transporter resembling Pgp (ABCB1) sensitive to verapamil (known inhibitor of human Pgp) was described in two bivalves species (*Anodonta cygnea* and *Mytilus galloprovinciallis*) (Kurelec and Pivčević, 1989, 1991). This study was the starting point for the identification and characterization of the MXR phenotype in several other species of aquatic organisms, such as sponges (Kurelec, 1992), molluscs (McFadzen et al., 2000; Minier et al., 2000; Smital et al., 2003; Luckenbach and Epel, 2008; Faria et al., 2011), crabs and sea urchins (Toomey and Epel, 1993; Hamdoun et al., 2002). In fish, the presence of ABC efflux transporters have been described for an increasing number of species, such as winter flounder (Chan et al., 1992), antarctic fish (Zucchi et al., 2010), rainbow trout (Zaja et al., 2008a; Fischer et al., 2010; Loncar et al., 2010), zebrafish (Long et al., 2011a,b; Fischer et al., 2013), mullet (Diaz de Cerio et al., 2012), killifish (Paetzold et al., 2009), nile tilapia (Costa et al., 2012b, 2013), catfish (Liu et al., 2013) and seabass (Ferreira et al., 2014) (Table 2).

**Table 2:** **ABC transporters involved the efflux of toxic compounds, fish species where they were identified and tissue distribution pattern in mammals and fish**.

**Protein**	**Mammals**		**Fish**			
	**Tissue distribution**	**Localization in polarized cell**	**Specie**	**Common name**	**GenBank accession number**	**Tissue distribution**
ABCB1	BBB, liver, intestine, kidney, placenta, stem cells	Apical	*Barbus barbus*	Barbel	DQ059069.1	Liver, intestine, kidney, brain, gonads^a^^,^^b^^,^^c^^,^^d^^,^^e^^,^^f^^,^^g^^,^^l^^,^^m^
			*Carassius auratus*	Goldfish	DQ059072.1	
			*Chelon labrosus*	Gray mullet	HM467814	
			*Chondrostoma nasus*	Nase	AY948951.1	
			*Cyprinus carpus*	Carp	AY999964.1	
			*Danio rerio*	Zebrafish	XP_001922717	
			*Dicenthrarchus labrax*	European seabass	GQ273979.1	
			*Fundulus heteroclitus*	Killifish	AF099732.1	
			*Leuciscus cephalus*	Chub	AY999966	
			*Mullus barbatus*	Red mullet	AY850375.1	
			*Oncorhynchus mykiss*	Rainbow trout	AY863423.3	
			*Oreochromis niloticus*	Nile tilapia	GQ911571	
			*Plactichthys flesus*	European flounder	AF175686.1	
			*Pleuronectes americanus*	Winter flounder	AY053461.1	
			*Poeciliopsis lucida*	Topminnow	DQ842514.2	
			*Scophtalmus maximus*	Turbot	AJ291813	
			*Trematomus bernacchii*	Rock cod	FJ938210.1	
ABCB11	Liver	Apical	*Chelon labrosus*	Gray mullet	HM467813	liver, intestine^a^^,^^l^^,^^m^
			*Danio rerio*	Zebrafish	XP_001923538	
			*Dicenthrarchus labrax*	European seabass	GQ273980.1	
			*Fundulus heteroclitus*	Killifish	AF135793.1	
			*Oncorhynchus mykiss*	Rainbow trout	DQ865266.2	
			*Oreochomis niloticus*	Nile tilapia	GQ911570	
			*Platichthys flesus*	European flounder	AJ344042.1	
ABCC1	Lung, testis, kidney, peripheral blood mononuclear cells, skeletal and cardiac muscle, placenta	Basolateral (apical in brain endothelial cells)	*Barbus barbus*	Barbel	FJ890350.1	testis, ovary, kidney, muscle, gills, heart, liver, intestine, brain, eye^a^^,^^h^^,^^l^^,^^m^
			*Chelon labrosus*	Gray mulet	HM467810	
			*Danio rerio*	Zebrafish	XM_002661202	
			*Dicenthrarchus labrax*	European seabass	GQ273982.1	
			*Oncorhynchus mykiss*	Rainbow trout	GQ166973.1	
			*Oreochromis niloticus*	Nile tilapia	GQ911567	
			*Oryzias melastigma*	Medaka	JN629038.1	
			*Plactichthys flesus*	European flounder	AJ344044.1	
			*Poeciliopsis lucida*	Topminnow	HM102361.1	
			*Trematomus bernacchii*	Rock cod	FJ938212.1	
ABCC2	BBB, liver, intestine, kidney, placenta, lung	Apical	*Carassius auratus*	Goldfish	FJ890349.1	liver, kidney, intestine, brain, muscle, gills^a^^,^^i^^,^^j^^,^^k^^,^^l^^,^^m^^,^^n^
			*Chelon labrosus*	Gray mullet	HM467815	
			*Chondrostoma nasus*	Nase	AY948950	
			*Cyprinus carpio*	Carp	AY679169	
			*Danio rerio*	Zebrafish	NM_200589.1	
			*Dicenthrarchus labrax*	European seabass	GQ273983.1	
			*Leuciscus cephalus*	Chub	FJ890348.1	
			*Mullus barbus*	Barbel	AY275434.1	
			*Oncorhynchus mykiss*	Rainbow trout	NM_001124655.1	
			*Oreochromis niloticus*	Nile tilapia	GQ911569	
			*Plactichthys flesus*	European flounder	AJ344045.1	
			*Poeciliopsis lucida*	Topminnow	HM102360.1	
			*Trematomus bernacchii*	Rock cod	FJ938211.1	
			*Raja erinácea*	Little skate	AF486830	
ABCC3	Adrenal gland, Intestine, Pancreas, Gallbladder, Placenta, Liver, Kidney, Prostate	Basolateral	*Chelon labrosus*	Gray mullet	HM467809	Liver ^a^,^m^
			*Oncorhynchus mykiss*	Rainbow trout	GQ888533	
			*Poeciliopsis lucida*	Topminnow	DQ842515.1	
ABCC4	Ovary, Testis, Kidney, Lung, Prostate	Apical, basolateral	*Danio rerio*	Zebrafish	NM_001007038.1	Liver^a^
			*Oncorhynchus mykiss*	Rainbow trout	BX911853	
ABCC5	Liver, Testis, Skeletal and Cardiac Muscle, Brain	Basolateral, apical	*Danio rerio*	Zebrafish	HQ161064	Liver^a^
			*Oncorhynchus mykiss*	Rainbow trout	GU079635	
ABCG2	BBB, placenta, liver, intestine, breast, stem cells	Apical	*Chelon labrosus*	Gray Mullet	HM467811.1	liver, kidney, gonads, intestine, gills^a^^,^^l^^,^^m^
			*Dicenthrarchus labrax*	European seabass	GQ273981.1	
			*Oncorhynchus mykiss*	Rainbow trout	EU163724.1	
			*Oreochromis niloticus*	Nile tilapia	GQ911568	
			*Poeciliopsis lucida*	Topminnow	HM102358.1	
			*Salmo salar*	Atlantic salmon	NM_011736655.1	

a Loncar et al., 2010;

b Kleinow et al., 2000;

c Doi et al., 2001;

d Bard et al., 2002a;

e Bard et al., 2002b;

f Klobučar et al., 2010;

g Miller et al., 2002;

h Long et al., 2011a;

i Long et al., 2011b;

j Miller et al., 2007;

k Cai et al., 2003;

l Costa et al., 2012b;

m Diaz de Cerio et al., 2012;

n Ferreira et al., 2014.

Identification of ABC transporters and characterization of the MXR mechanism has been achieved by the use of several detection methods, including quantitative or semi-quantitative reverse transcription polymerase chain reaction (qRT-PCR or RT-PCR) and *in situ* hybridization to evaluate mRNA expression of genes, immunochemical techniques for protein detection applying mammals monoclonal antibodies to ABCB1 (western blotting or immunohistochemistry), northern-blotting and activity assays (through the measurement of efflux or accumulation of fluorescent model substrates).

Most studies directed to the determination of organ distribution pattern of ABC transporters in aquatic organisms have been directed to Pgp by the use of the mammalian anti-Pgp monoclonal antibody (mAb) C219. This mAb which recognizes an epitope common to all known Pgps (in human MDR1 and MDR3) and also to Spgp (ABCB11) (Georges et al., 1990; Childs et al., 1995; van den Elsen et al., 1999). After probing with the mammalian mAb C219, a protein related to the mammalian MDR protein was identified in embryos and gills of a few aquatic invertebrates (Toomey and Epel, 1993; Cornwall et al., 1995). In fish, positive reaction was seen in the hepatic bile canaliculi (Kleinow et al., 2000; Doi et al., 2001; Bard et al., 2002a,b; Klobučar et al., 2010; Costa et al., 2013), apical membrane of enterocytes (Kleinow et al., 2000; Doi et al., 2001; Bard et al., 2002a; Costa et al., 2013), kidney (Kleinow et al., 2000), and in endothelial capillaries of brain (Miller et al., 2002). A negative reaction of the mAb C219 was patent in gills, kidney, gonad, brain, spleen, heart of killifish and blenny (Bard et al., 2002a,b). In summary, the suggested Pgp localization in fish reveals a pattern similar to that described in mammals, with immunoreactive Pgp occurring in epithelial tissues involved in secretion, absorption or serving a barrier function, For other ABC efflux transporters no fish-functional antibodies are available, thus information on their distribution pattern is quite limited and restricted to the mRNA expression among the various fish organs.

*ABCB1* expression has been detected in different fish species, and in distinct organs as described in Table 2. These evidences point to the important role that ABCB1can have also in fish species validating the research on this Pgp-like proteins. As in mammals, ABCB11 protein was found to be almost exclusively expressed in liver, with low expression in proximal intestine, and very low expression in other organs as gills, brain, gonads and kidney of trout (Loncar et al., 2010), in trout cell lines (Fischer et al., 2011) and nile tilapia (Costa et al., 2012b), supporting its role in the efflux of bile salts from hepatocytes into the bile. For ABCCs the information available is more limited and efforts in the last years have increased the information available. In trout, low *ABCC1* expression was found, and kidney was the only organ showing notable *ABCC1* expression while significant expression levels of *ABCC2* in major detoxification organs were reported (Loncar et al., 2010). In nile tilapia, *ABCC1* and *ABCC2* were shown to be expressed in adult organs like liver, gill and intestine (Costa et al., 2012b) and also in early developmental stages of the larvae (Costa et al., 2012a). *ABCC1* and *ABCC2* were also present in European seabass liver and responsive to a PAH (Ferreira et al., 2014). High *ABCC2* expression was previously reported in kidney, intestine and liver of little skate (Cai et al., 2003) and in apical membrane of proximal kidney tubule of killifish (Miller et al., 2007). Regarding ABCG2, Loncar et al. (2010) have examined the organ distribution pattern of this protein in trout, and found high expression levels in gonads and moderate expression in distal part of intestine, kidney and brain: previously, expression of *ABCG2* was also reported by the same group in trout liver and primary hepatocytes (Zaja et al., 2008b) (Table 2). Also the studies performed in our group with nile tilapia have shown that *ABCG2* is expressed in barrier organs and at different development stages (Costa et al., 2012a,b).

Based on these results and on mammal studies, investigators believe that, in aquatic organisms, ABC efflux transporters should follow a similar distribution pattern and cellular localization, with expression in organs involved in secretion, absorption or serving as a barrier function, like liver and intestine. Thus, ABCB1, ABCC2, and ABCG2 should be localized in the apical membrane of polarized cells, pumping substrates into the intestinal lumen or bile canaliculi, while ABCC1 localization in basolateral membrane results in the export of its substrates into the blood. Additionally, ABCB1 should efflux mostly unmodified compounds, while ABCCs and ABCG2 should efflux organic anions conjugated by phase II enzymes (Epel et al., 2008).

The bulk of studies conducted in aquatic organisms to evaluate the interaction of environmental contaminants with ABC efflux transporters have been mostly performed on Pgp. Some compounds, as Pgp model reversal agents (verapamil and/or cyclosporine A) and xenobiotics (like the insecticide malathion) were shown to inhibit Pgp activity in fishes, resulting in an increase of the bioaccumulation of toxic compounds like 2-aminoanthracene, hydrocarbon-rich Diesel-2 oil, and/or model Pgp substrates like rhodamine B and rhodamine 123 (Smital and Kurelec, 1998; Fischer et al., 2013; Ferreira et al., 2014). Other studies have demonstrated the ability of some xenobiotics to induce Pgp expression in fish, such as cadmium (Zucchi et al., 2010), the organophosphate insecticide chloropyrifos and the carcinogen N-nitrosodiethylamine (Albertus and Laine, 2001). Similarly, resistance-killifish populations from highly polluted sites also demonstrated enhanced Pgp expression (Cooper et al., 1999; Bard et al., 2002a) suggesting a protective role of Pgp. Nevertheless, in another study performed in killifish from a heavily polluted site with polycyclic aromatic hydrocarbons (PAHs), polychlorinated biphenyls (PCBs) and heavy metals, Paetzold et al. (2009) found no up-regulation of hepatic *ABCB1* transcripts. Similarly, after *in vivo* exposures of fishes to different xenobiotics, such as prochloraz, nonylphenol diethoxylate, ß-naphthoflavone, BaP and 3,4,3',4'-tetrachlorobiphenyl no changes were seen in the levels of hepatic and/or intestinal Pgp (Doi et al., 2001; Sturm et al., 2001; Costa et al., 2012b, 2013). The study of Pgp in fish species have been given some contradictory results. A recent study in zebrafish revealed that *ABCB4* acted as the functional multixenobiotic transporter and showed activity as a barrier to chemical uptake (Fischer et al., 2013). Also in seabass the difficulty to assess *ABCB1* expression (Ferreira et al., 2014) can be an indication of different transporters to be functional in different fish species.

Few studies have addressed the response of other ABC efflux transporters upon animals' exposure to pollutants in comparison to Pgp. Paetzold et al. (2009) described an up-regulation of hepatic transcripts for *ABCC2* and *ABCG2* in a multiresistant population of killifish living in a pond heavily polluted with PAHs, PCBs and heavy metals, while no up-regulation was seen in *ABCB1* and *ABCB11* mRNA. Exposure to metals has induced the expression of *ABCC2* in excretory organs of zebrafish including kidney, liver and intestine (Long et al., 2011b) and also Cd-exposed *Trematomus bernachii* (Zucchi et al., 2010). Moreover, an up-regulation of *ABCC2* was seen in red mullet liver from an oil contaminated site (Della Torre et al., 2010). Long and co-workers described an up-regulation of *ABCC1* gene in ZF4 cells and embryos of zebrafish exposed to heavy metals (Long et al., 2011a,b). Increased expression of *ABCC2* was observed in seabass primary hepatocytes (Ferreira et al., 2014) and in Nile tilapia gill after waterborne exposure to BaP (Costa et al., 2012b). *ABCG2* expression was also up-regulated in Nile tilapia intestine upon BaP exposure (Costa et al., 2012b). These recent studies on MRPs and BCRP highlight the important role these ABC transporters may play in the overall detoxification process and cooperation with phase I and II biotransformation enzymes. Nevertheless the contribution of the different ABC proteins in the efflux of xenobiotics has to be conclusively proved.

Although an increased number of compounds have been shown to interact with ABC efflux transporters, data remains fragmented and largely restricted to laboratory observations in comparison to relevant field data. It remains uncertain which classes of environmental toxicants can induce and/or repress the activity of these proteins, and to what extent this MXR mechanism protects aquatic animals from the toxic action of xenobiotics. There is a fundamental need to identify the common features of ABC efflux transporters substrates, but also the characteristics of chemicals that might inhibit their activity leading to an increased toxicity of normally effluxed chemicals. Most ABC efflux transporters are remarkably non-specific with respect to its substrates. This feature can be an important advantage, as it provides protection against many classical and novel anthropogenic products, but it can also render the system vulnerable (Smital et al., 2004; Epel et al., 2008). The presence of multiple substrates that compete for binding sites can saturate these locations, or directly inhibit the transporter activity, in a phenomenon termed chemosensitization (Bard, 2000; Epel et al., 2008). MXR inhibitors, or chemosensitizers, were divided in two main classes: (1) competitive inhibitors with high affinity that prevent the binding and active transport of other substrates; and (2) non-competitive inhibitors that can act in different ways, such as blocking ATPase activity (Smital et al., 2004). The presence of MXR inhibitors can compromise the effectiveness of the defense system, since toxic substances that would normally be excluded, will remain in the cell and exert their toxic effects (Epel et al., 2008; Fischer et al., 2013). Ability to inhibit ABC transporters of aquatic organisms has been described for different types of contaminants such as PPCPs, pharmaceuticals, pesticides, fragrances, among others (Bard, 2000; Smital et al., 2004; Epel et al., 2008; Fischer et al., 2013), however we still do not know the actual consequences that chemosensitization will have at population and ecosystem level. Progress in research of these proteins has been delayed mostly due to the existence of technical limitations, as, for example, the inexistence of specific probes/antibodies for these transporters in aquatic organisms, and the measurement of its functionality. Thus, there is the need to further investigations in the MXR mechanism in order to fully understand the physiological and toxicological functions of ABC transporters in aquatic organisms, including fish.

## From phase 0 to phase III—A complete pathway for cellular detoxification

Biological membranes are barriers for the uptake, distribution and elimination of xenobiotics in the organism (Simkiss, 1995). Bioaccumulation of organic compounds occurs mainly passively, driven by the compound's hydrophobicity, and once absorbed, the xenobiotic may undergo a detoxification pathway, in order to be excreted from the organism before it exerts toxic effects (Van Der Oost et al., 2003). The most studied pathway for cellular detoxification is the biotransformation of xenobiotics by phase I and phase II enzymes. Biotransformation consists on a two-phase process of enzymatic reactions that alter the chemistry of non-polar lipophilic chemicals to polar-water soluble metabolites, leading to the detoxification and elimination of the parent compound (Black and Coon, 1987; Buhler and Williams, 1989). The enzymes of phase I metabolism (oxidation, reduction, hydration, hydrolysis) introduce a functional group (−OH, −COOH, −NO2, etc.) into the xenobiotic (Commandeur et al., 1995). Phase II of the biotransformation process involves the conjugation of the xenobiotic parent compound or its phase I metabolites with an endogenous ligand, thus facilitating the excretion of chemicals by the addition of more polar groups [e.g., glutathione (GSH), glucuronic acid (GA), sulfate] (Schlenk et al., 2008). In recent years it has been proposed that two additional steps of drug disposal, called phase 0 and phase III, are equally important as the biotransformation process (Szakács et al., 2008). These phases involve the modulation of the cellular efflux, by ABC transporters, of either unmodified or metabolized compounds and xenobiotic efflux transporters and biotransformation enzymes may be part of a coordinated defense mechanism that protects cells from xenobiotic insults (Xu et al., 2005). Thus, understanding the underlying facts behind the possible cooperative action of efflux transporters and biotransformation enzymes has critical implications both for human and environmental health. However, despite its relevance, studies focused on the mechanisms important for this coordinated defense that protects cells from xenobiotic insults in non-mammalian models, including fish, are still very scarce (Bard, 2000). Moreover, the studies published so far did not provide a consistent pattern on the cooperation mechanisms, mostly due to the insufficient knowledge of the basic parameters of ABC transcriptional regulation in fish. Intracellular receptors are proteins which, upon binding of specific ligands, have the ability to regulate the mRNA transcription of specific genes, thereby controlling development, homeostasis, and metabolism of the organism (Xu et al., 2005). From mammalian models, several receptors (e.g., Aryl hydrocarbon receptor-AhR, nuclear factor-erythroid 2 p45-related factor-Nrf2, pregnane X receptor-PXR, peroxisome proliferated activated receptor-PPAR, liver X receptor-LXR, farnesoid X receptor-FXR, retinoid X receptor-RXR, constitutive androstane receptor-CAR, glucocorticoid receptor-GR) have been shown to be key mediators in the regulation of the activity of phase I and phase II metabolizing enzymes, and ABC transporters involved in efflux mechanisms (Xu et al., 2005). Some studies in mammalian *in vitro* models have associated some nuclear receptors to the regulation of expression of ABC transporters (Naspinski et al., 2008; Chisaki et al., 2009; Rigalli et al., 2011), such as the induction of expression of phase I and mdr genes via CAR and PXR pathways (Gu et al., 2013; Wang et al., 2013a,b). Even though in fish there is a fundamental lack of knowledge on the transcriptional regulation of ABC transporters, in zebrafish an association was shown between PXR, CYP3A and MDR1 (Bresolin et al., 2005), and the same pathways of transcriptional regulation of detoxification enzymes and transporters as in mammals are expected.

However, whether phase I and II enzymes inducers co-ordinately regulate ABC efflux transporters genes requires further studies that can shed some light on the role of receptors, transcription factors and signaling cascades of this metabolism/transport of endogenous and exogenous compounds (Xu et al., 2005). Also in mammalian studies, induction of Pgp has been reported after exposure to known inducers of phase I (CYP1A) and phase II enzymes, like BaP and 3-methylcholanthrene (3MC) (Chao Yeh et al., 1992; Fardel et al., 1996). Moreover, ABCC1, ABCC2 and ABCG2 are known to efflux mainly phase II metabolites, xenobiotics conjugated with GSH, GA and/or sulfate in mammalian models (for a review see Leslie et al., 2005), further supporting their role in phase III of the detoxification process.

**Figure 3:**
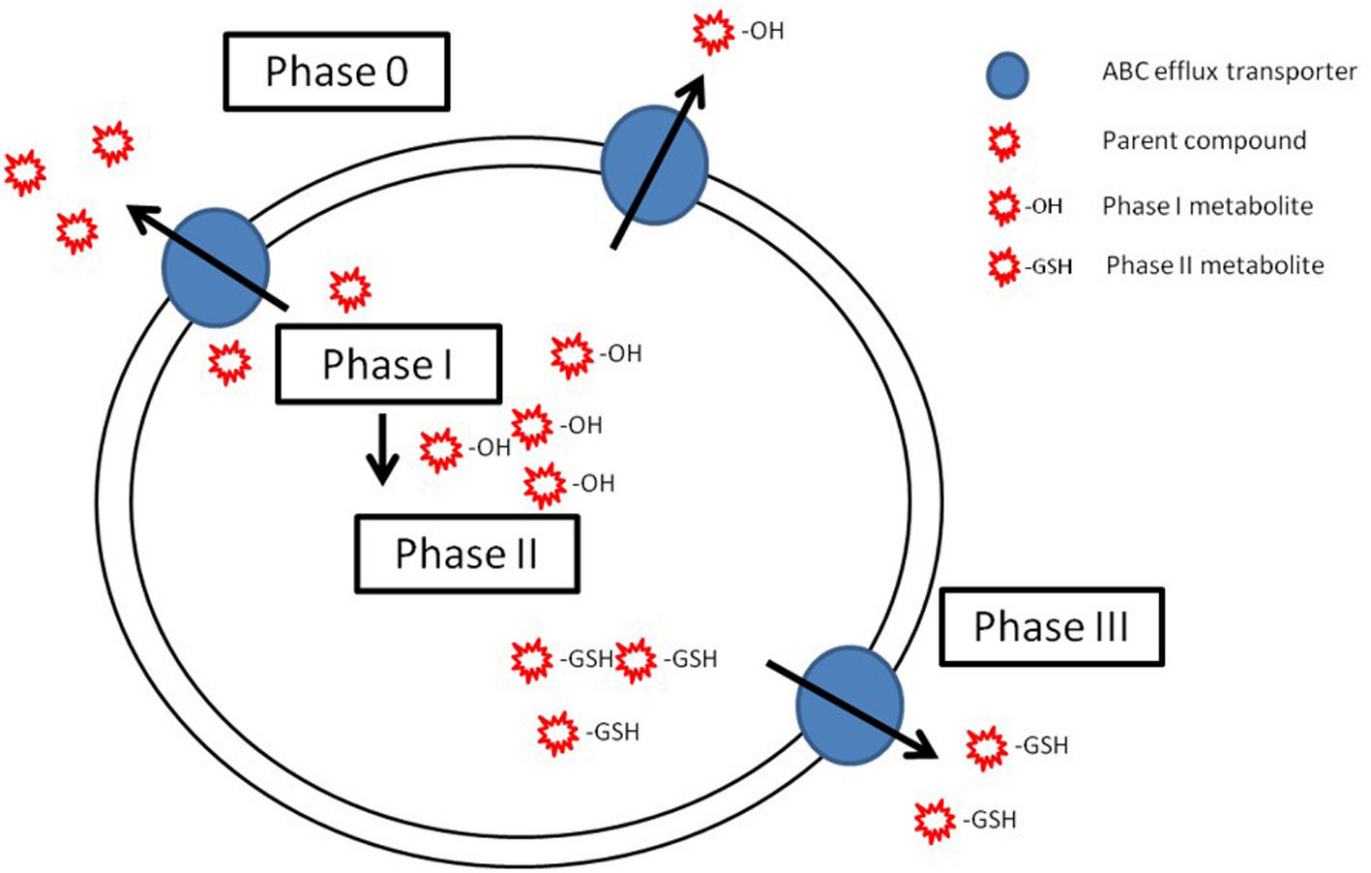
**Schematic representation of the possible cooperation of ABC efflux transporters (phase 0 and phase III) and biotransformation enzymes (phase I and phase II) in cellular detoxification**. In phase 0, ABC transporters (blue circles) efflux the parent compounds (red stars). Parent compound are biotransformed by phase I in metabolites (red star with an −OH) that are conjugated on phase II (red stars with −GSH). Adapted from Bard (2000), with permission.

Considering the characteristics of common regulation mechanisms and complementarity of substrates, it is believed that ABCB1 acts as a first line of defense preventing unmodified compounds from accumulating in the cell (phase 0 of cellular detoxification), while ABCCs and ABCG2 transport phase I and II metabolites, therefore acting in phase III of cellular detoxification (Bard, 2000; Sturm and Segner, 2005; Epel et al., 2008) (Figure 3). Compared to biotransformation enzymes, research on efflux transporters is still very limited and has largely been confined to mammalian models. In aquatic organisms, a few studies have been conducted in an attempt to elucidate this cooperative mechanism (Cooper et al., 1999; Sturm et al., 2001; Bard et al., 2002b; Paetzold et al., 2009; Costa et al., 2012b; Ferreira et al., 2014). The majority of these studies have been confined to the study of Pgp and CYP1A, and results suggest that these two proteins should not be coordinately regulated in fish, but enhance the fact that they may act in complementarity in cellular detoxification. More recently, this cooperative role has been pointed out in nile tilapia exposed to benzo(a)pyrene by showing increase in expression of both phase I and phase II biotransformation enzymes as well as the toxicological relevant efflux proteins in barrier organs (Costa et al., 2012b, 2013). However, the database on the relation between Pgp and biotransformation enzymes in fish is small and dispersed, and the role of other ABC efflux transporters, besides Pgp, is even more poorly documented. Thus, the full characterization of this mechanism of cellular detoxification, where ABC efflux transporters and biotransformation enzymes display title-roles is yet far from being completely understood.

## Conclusions

Understanding the underlying facts behind the mechanisms of cellular detoxification has implications both for human and environmental health. The medical relevance of these transporters is evidenced by their MDR abilities and, under this point of view there is a fundamental need to know how to properly block the action of these transporters to assure the targeting efficacy of chemotherapeutic drugs. In addition, the study of ABC efflux transporters is also very important from an environmental point of view, since they are believed to be an integral part of the cellular detoxification system. For environmentalists, the goal is to understand how these efflux transporters keep toxicants out of the cells, and to ensure that they operate optimally in order to protect the cells from environmental contaminants. The effects of ABC transporters on the toxicity of environmental contaminants is still poorly understood, thus efforts must be made to provide insights into the interactions of contaminants in fish species. In particular, interactions with classical and emergent compounds and the environmental relevance of fish ABC transporters is yet to be thoroughly addressed. The search for chemosensitizers among emergent compounds is relevant since some contaminants considered to be weak or moderately toxic at concentrations found in the aquatic environment can be potent inhibitors of ABC transporters and modulate toxicity of other xenobiotics and led to unpredicted effects to exposed species. Therefore, and in particular with emergent compounds, it is important to evaluate interaction of a large array of contaminants with ABC transporters when assessing toxicity of xenobiotics to the aquatic environment. One difficulty to fully address the role of ABC transporters in the detoxification process and the interactions with xenobiotics with the different classes of contaminants is that the information available on fish species is dispersed in different studies with different goals. So in the future, research must be directed to the study of regulation of ABC transporters and to unravel the importance of these efflux proteins to the overall maintenance of the homeostasis of fish species chronically exposed to foreign chemicals.

### Conflict of interest statement

The authors declare that the research was conducted in the absence of any commercial or financial relationships that could be construed as a potential conflict of interest.
